# Recent Advances in Green Metallic Nanoparticles for Enhanced Drug Delivery in Photodynamic Therapy: A Therapeutic Approach

**DOI:** 10.3390/ijms24054808

**Published:** 2023-03-02

**Authors:** Alexander Chota, Blassan P. George, Heidi Abrahamse

**Affiliations:** Laser Research Centre, Faculty of Health Sciences, University of Johannesburg, P.O. Box 17011, Johannesburg 2028, South Africa

**Keywords:** cancer, nanoparticles, photosensitizer, theranostic, photodynamic therapy

## Abstract

Globally, cancer is one of the leading causes of death among men and women, it is characterized by the unregulated proliferation of tumor cells. Some of the common risk factors associated with cancer development include the consistent exposure of body cells to carcinogenic agents such as alcohol, tobacco, toxins, gamma rays and alpha particles. Besides the above-mentioned risk factors, conventional therapies such as radiotherapy, and chemotherapy have also been linked to the development of cancer. Over the past decade, tremendous efforts have been invested in the synthesis of eco-friendly green metallic nanoparticles (NPs), and their medical application. Comparatively, metallic NPs have greater advantages over conventional therapies. Additionally, metallic NPs can be functionalized with different targeting moieties e.g., liposomes, antibodies, folic acid, transferrin, and carbohydrates. Herein, we review and discuss the synthesis, and therapeutic potential of green synthesized metallic NPs for enhanced cancer photodynamic therapy (PDT). Finally, the advantages of green hybridized activatable NPs over conventional photosensitizers (PSs) and the future perspectives of nanotechnology in cancer research are discussed in the review. Furthermore, we anticipate that the insights offered in this review will inspire the design and development of green nano-formulations for enhanced image-guided PDT in cancer treatment.

## 1. Introduction

The term cancer refers to a group of medical conditions in which body cells proliferate in an unregulated manner. If not treated and well managed, cancer may lead to the development of life-threatening medical complications that may lead to death. According to the GLOBOCAN 2020 report, cancer has been ranked as the major cause of mortality among men and women. Globally, the global incidence of cancer is projected to rise by 66.7% by the year 2040. Moreover, many studies have reported that age, alcohol, genetic mutations, and smoking are some of the predisposing risk factors that promote cancer development. Recently, co-morbid infections such as human immunodeficiency virus (HIV), human papillomavirus (HPV), and severe acute respiratory syndrome coronavirus 2 (SARS-CoV-2) have also been reported to directly or/indirectly promote the development of cancer. In addition, current and conventional therapeutic modalities used in treating cancer include radiotherapy, surgical resectioning, chemotherapy and targeted therapies. Despite the aforementioned advantages associated with conventional therapies, these therapies are directly or indirectly linked with cancer resistance and recurrence at the advanced stages of disease progression [1]. Therefore, this warrants the exploration of other therapeutic modalities with minimised side effects e.g., photodynamic therapy. 

Photodynamic therapy (PDT) is a non-invasive clinically approved localized form of therapy that uses non-ionizing radiation to induce tumor cell death via intracellular generation of reactive oxygen species (ROS). Besides its use of non-ionizing radiation, the hallmark of this therapy dually depends on tumor cell selectivity and preferential intracellular co-localization of a photosensitizer (PS) within organelles of tumor cells such as the mitochondrion, lysosomes, and endoplasmic reticulum. Furthermore, its mechanism of action and efficacy in cancer therapy sorely depends on the interaction of three major components i.e., non-ionizing radiation, PS, and molecular oxygen (O_2_). However, recent studies report conventional PDT as having therapeutic limitations such as non-specificity, light dose, wavelength, and fluence rates [2]. In order to avoid the above highlighted limitations, many and recent research projects have diverted their focus from direct use of conventional PS’s in PDT via the design of novel photochemical molecules that are incorporated with nanomaterials e.g., liposomes for enhanced drug delivery through the use of nanotechnology. Although nanotechnology is a widely studied form of science with applications ranging from computing, environmental science, and medicine. Green nanotechnology is a novel therapeutic modality that employs the use of oxidizing agents e.g., silver nitrate (AgNO_3_) salts, PS’s, plant extracts or/plant derived bioactive compounds etc., to synthesize therapeutic green nanoparticles (NPs) with enhanced therapeutic effects in PDT. Herein, this review highlight, and discuss the most employed drug delivery avenues for NPs in cancer therapy. Thereafter, we discuss the frameworks associated with the photophysical and photochemical processes of green nano-formulation behind the mechanism behind PDT in cancer therapy.

## 2. Fundamental of Photophysics and Photochemistry of PDT

**Figure 1:**
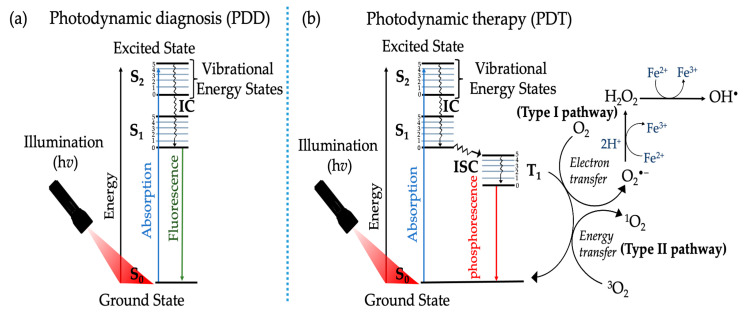
Modified Jablonski energy diagram depicting the underlying photophysical and photochemical reactions of PSs in photodynamic diagnosis (PDD) (**a**) and photodynamic therapy (PDT) (**b**).

The mechanism behind PDT depends on the presence of light at a specific wavelength (λ), molecular O_2_, and light absorbing PS to generate molecular cytotoxic ROS used in the eradication of various medical alignments including cancer [3]. As well illustrated by the modified Jablonski energy diagram (Figure 1), the modified illustration shows a sequential series of photophysical and photochemical events that lead to the generation of intracellular cytotoxic ROS post light absorption of preferentially intracellularly co-localized PS in tumor cells. PS excitation occurs when intracellularly co-localized PS interacts with photon energy and biomolecules possessing electromagnetic energy transitions that are equal to those of the photon, thus promoting the excitation of ground singlet (S_0_) PS’s to an activated and excited electronic state known as the singlet (S_1_) state. More precisely, two possible processes occur once the PS is at the S_1_ state, the first possibility involves the movement of the S_1_ state to the S_0_ state thus emitting photon energy in form of fluorescence. It is worth mentioning that this type of photophysical process is mostly used for photodynamic diagnosis (PDD) (Figure 1a). The second possibility of the S_1_ state going to the S_0_ state involves valence electron rearrangements in spins accompanied by intersystem crossing (ISC), triplet (T_1_) formation, internal conversion (IC), and phosphorescence. More importantly the T_1_ state has a longer half-life when compared the S_1_ state of an excited PS. In the presence of biomolecules or/T_1_ state molecules such as ^3^O_2_, the excited T_1_ state of the PS may trigger the induction of photochemical reactions, behind the rationale of PDT (Figure 1b).

In addition to the aforementioned photophysical processes, there are two major distinct photochemical pathways (i.e., Type I and Type II) that upon activation lead to free radical and ROS generation post PDT (Figure 1). The mechanism behind the Type I photochemical pathway depends on the induction of reduction and oxidation (redox) reactions. During this process, electrons or/hydrogen ions are transferred between excited T_1_ state of a PS and adjacent biomolecules, thus owing to the generation of superoxide anions (O_2_^•–^) or free radicals e.g., hydroxyl radicals (OH^•^), and hydrogen peroxide (H_2_O_2_). Alternately, Type II photochemical pathway depends on the transfer of energy from the excited T_1_ state PS to the ground T_1_ state ^3^O_2_, hence leading to cytotoxic singlet oxygen (^1^O_2_) generation. Further, the photochemical effects of these two pathways in PDT mainly depends on the ratios of the PS used, availability of substrates, and ^3^O_2_ [3]. It is also worth mentioning that these two pathways may occur simultaneously in a competitive manner. 

Despite the continued use of numerous conventional dyes and PS’s for cancer diagnostic and therapeutic purposes, extensive studies report these chemicals having poor solubility and stability, high doses for diagnostics and therapeutics, low ^1^O_2_ quantum yield, low selectivity, longer elimination time accompanied by skin photosensitivity [4]. Argumentatively PSs for diagnostic and therapeutic purposes must possess a higher chemical purity, increased desolvation properties, specificity for targeted body cells or/tissues, longer T_1_ state lifetime, and a higher ^1^O_2_ quantum yield post illumination. In addition to the above-mentioned chemical properties, ideal dyes and PSs must have minimal dark toxicity effects post illumination at an appropriate λ. To improve aforementioned phototherapeutic properties of conventional PSs used in cancer therapy, different classes of PSs (i.e., first, second, and third generation PSs) may either be used as conjugate for a nanocarrier or as a mixture with different nano-formulation carriers e.g., lipids, carbon nanotubes etc. [5]. Table 1 provides a summary on the advantages and disadvantages of photodynamic therapy as well as current conventional cancer therapies while Table 2 gives a summary on the current classification, photophysical and photochemical properties as well as the clinical indications/ applications of clinical PSs.

## 3. Nanoparticles

Nanoparticles (NPs) are very small/ tiny molecules with structural dimensions measured on the nanoscale i.e., diameter in the range between 1 nm to 100 nm in size [19]. These particles can be broadly categorized into a number of groups based on their morphological, structural size, photophysical and chemical characteristics [19,20,21]. In recent years, the use of NPs in the medicinal industry has drastically increased, this is because of their smaller size in nature, which enables them to easily penetrate cellular plasma membranes (i.e., via the use of cellular endocytic mechanisms of cells) [22,23]. Additionally, NPs have significantly emerged as essential key players for enhanced drug delivery systems in modern medicine [24]. Further, NPs can also be functionalized with different therapeutic/ or targeting moieties for active cellular targeting (e.g., liposome encapsulation of hydrophobic and hydrophilic antibiotics, photosensitizing agents, and anticancer drugs) [25]. Over the past years, many nanomaterials as well as nanocarriers have been studied for their anticancer properties. Examples of such materials are covered and summarised in Table 3. Despite the aforementioned characteristics, advantages, medical applications, and advancements of NPs in cancer diagnosis and therapy, conventional nano-based anticancer therapies continue to induce undesired photochemical and immunotherapeutic effects (e.g., acute and chronic inflammatory responses) i.e., if NPs are not well functionalized for therapeutic purposes [26,27]. Moreover, the applications, safety, and biosecurity of NPs in a clinical setup is not well understood. Preclinical and clinical studies still suggest a thorough analysis of NPs before their routine therapeutic use [28,29,30]. Herein, we discuss the benefits of the use of plant derived-bioactive compounds as a safe and novel source of therapeutic compounds for enhanced anticancer efficacy in PDT. 

### Plasmonic Photothermal Effects of Metallic NPs in PDD and PDT 

**Figure 2:**
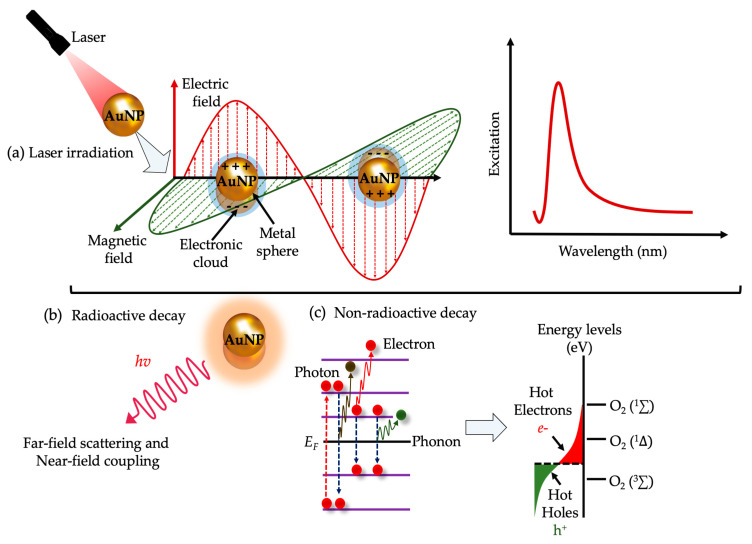
Schematic representation of surface plasmon resonance (SPR) of metallic nanoparticles (NPs). Photoexcitation and relaxation of plasmonic NPs generate localized surface plasmon resonance (LSPR) (**a**–**c**). Post-irradiation, plasmonic NPs may undergo two major processes, i.e., radioactive decay /or non-radioactive decay (**b**,**c**). Radioactive decay can either lead to far-field light scattering into adjacent nanostructures, or/electromagnetic fields, i.e., near-field (**a**,**b**). Alternatively, LSPR can undergo a non-radioactive decay process in which intraband or/interband excitation levels within the conduction band of a metallic NP can result in the generation of hot electrons (**c**). The interactions of high energy hot electrons with molecular oxygen (O_2_) can result in the generation of cytotoxic reactive oxygen species (ROS). Further, the electronic energy states of hot electrons and holes are depicted by the colors red and green respectively (**c**).

Surface plasmon resonance (SPR) is an optical analytical technique used to study, and measure the interactions of different molecules and electrons in real time [59,60]. SPR may occur when a specific photon of incident light hits the surface of a metallic particle (e.g., copper, gold, silver, etc.) [61]. Upon irradiation of a metallic particle at a specific λ and incident angle, a portion of electric field propagating light energy may interact with the metallic coating and free electrons found on the surface of the metal, thus leading to free conducting band electron oscillation [62,63]. In PDD and PDT, the collective oscillation motion of free electrons can lead to electron scattering, fluorescence, phosphorescence, formation of O_2_^•–^, alternatively the formation of cytotoxic ^1^O_2_. A summarized schematic representation of the SPR in PDD and PDT is illustrated in Figure 2.

## 4. Green Nanotechnology in Cancer Treatment 

Medicinal plants, also referred to as medicinal herbs, have been studied and used in traditional medicine practices for many years (e.g., African folk medicine, traditional Chinese medicine, and traditional Indian medicine) to treat different medical conditions and ailments. It is also worth mentioning that plants synthesize hundreds of bioactive compounds as well as secondary metabolites which may be classified as alkaloids, flavonoids, tannins, saponins, and phenolic acids. In addition, retrospective studies have demonstrated that these compounds may be used as reducing and stabilizing agents, thus preventing non-hazardous aggregation and agglomeration of metallic NPs [64]. Despite the fact that the exact mechanisms underlying the biosynthetic processes of green metallic NPs are not yet apparent, a number of studies propose that metallic NPs are formed through the bio-reduction method e.g., gold (Au), and silver (Ag) NPs [64,65]. Furthermore, the use of green synthesized NPs for cancer diagnostics and therapeutics has increased over the past decade. This can be attributed to the abundant number of phytocompounds with therapeutic properties contained within medicinal plants.

Although green nanotechnology is relatively new, this branch of technology was designed to synthesize not only environmentally friendly products, but also to synthesize biocompatible, and chemically inert products for therapeutic purposes. Secondly, green nanotechnology by-products can also be incorporated with conventional NPs, thereby reducing some of the limitations associated with conventional NPs e.g., environmental hazards, high dose, and undesired toxicity post therapy. Besides the above therapeutic applications of green nanotechnology, green synthesized NPs can also be incorporated with inefficient metallic compounds, photosensitizing agents, imaging dyes, biocompatible nanocarriers e.g., liposomes, and targeting moieties such as monoclonal antibodies and nano-formulations. Additionally, the synthesis of green NPs is circumvent cost-effective and well stabilized when compared to the above mentioned inorganic conventional NPs highlighted in Table 3 [66,67].

**Figure 3:**
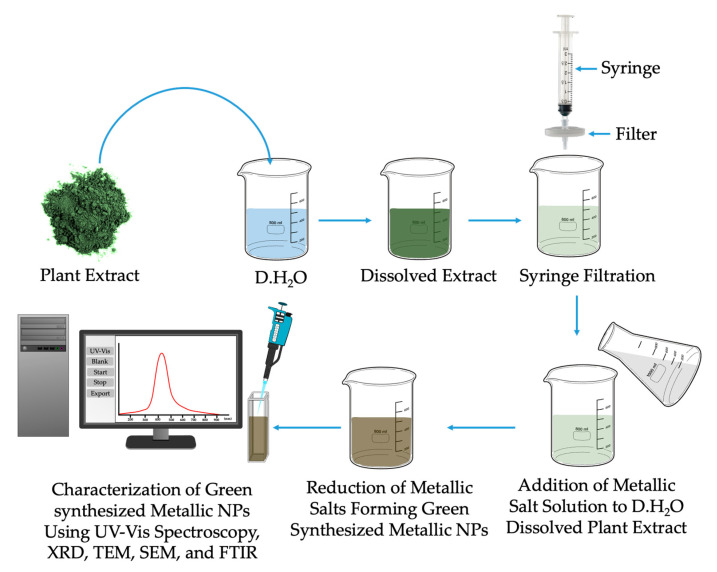
Schematic representation of the synthesis and characterization of green metallic nanoparticles.

Currently, there are different strategies employed in the synthesis of green NPs for diagnostics as well as theranostic purposes. These strategies may include biological, chemical, physical, and hybridization methods [68]. Although chemical methods are widely used in the synthesis of NPs, their application in clinical studies is limited. This is mostly attributed to the continued use of toxic chemicals and compounds that may not be eco-friendly. However, it is of utmost importance that eco-friendly methods such as the biological approaches where green NPs may be encapsulated, using highly biocompatible and biodegradable nanocarriers e.g., liposomes, pro-liposomes, and nano-formulations. Prior to the synthesis of green NPs, a plant with medicinal properties has to be identified, collected, washed thoroughly by using tap water, and later shade dried. After shade-drying, the part of interest of a plant (i.e., leaves, stem, or roots) has to be ground into fine powder, weighed, and subjected to extraction by using water and organic solvents (e.g., chloroform, ethanol, methanol, and etc.). Post extraction, the extracts need to be collected by evaporation of drying methods, prepared by desolvation in solvents (distilled water, or phosphate-buffered saline) and filtered by use the appropriate sterile filter (Figure 3). The collected plant filtrate may then be used to synthesize green metallic NPs, encapsulated with nanocarriers that are not loaded or not loaded with hydrophobic drugs. It is of great importance to mention that the extraction process, collection process, and green metallic NPs synthesis protocols are not only limited the above highlighted methods.

In addition, the formation of NPs may be confirmed and characterized by UV–visible (UV-Vis) spectrophotometry, dynamic light scattering (DLS), energy-dispersive spectroscopy (EDS), Fourier transform infrared (FTIR) spectroscopy, scanning electron microscopy (SEM), transmission electron microscopy (TEM), and X-ray diffraction (XRD) [69,70,71]. Table 4 represents an overview employed in the synthesis and characterization of green metallic NPs for cancer therapeutics.

### 4.1. Encapsulation Framework for Green Metallic NPs

Encapsulation is one of the common drug delivery systems adopted by many pharmaceutical companies. This approach tend to form a shell like protective layer for conventional agents (i.e., for delivery of hydrophilic or hydrophobic drugs), and its main role is to confine these drugs into a vesicle of the spherical structure, thereby preventing the leaching out of imaging dyes, and therapeutic drugs before they reach the targeted sites [86]. Although INPs and ONPs mentioned in Table 3 provide several benefits for the pharmaceutical sector which ranges from NP size, physicochemical properties, and medical applications e.g., drug delivery systems, bioimaging, theranostic, and therapeutics. The selectivity, biocompatibility, mechanisms of pharmacokinetic interactions, and cytotoxicity of most nanocarriers/or nano materials discussed in Table 3 are still unclear. Currently, liposomes are far much better than other drug delivery systems, this is because of liposomes are highly biocompatible, and easily biodegraded with minimized cellular toxicity. Since liposomes are self-assembling closed colloidal structures composed of phospholipid bilayers, their surface area can easily be functionalized by PEGylation (PEG) with different functional groups (e.g., amines, alcohols, carboxylic, aldehydes, esters, and thiol-derivatized ligands), and targeting ligands such as peptides, antibodies, or aptamers [87,88,89]. In addition, recent and modern liposomal drug encapsulation strategies allow the effective packaging of both hydrophilic and hydrophobic drugs for theranostic and therapeutic purposes, thus owing to the reduction of long-term systemic toxicity associated with conventional chemotherapeutic drugs such as doxorubicin [64,90].

**Figure 4:**
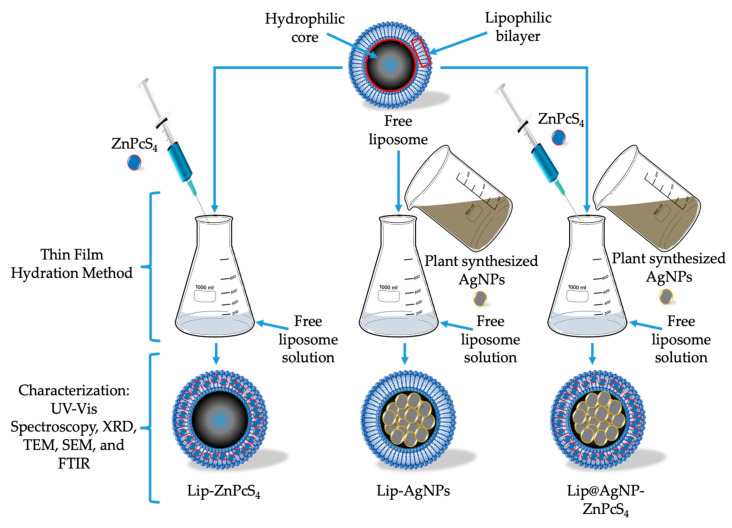
Schematic diagram representing green synthesis of Lip-ZnPcS_4_ and characterization techniques.

Doxorubicin (Dox) is a chemotherapeutic drug that is derived from the soil fungus *Streptomyces peucetius*, it is widely used in the treatment of solid tumors such as triple-negative breast cancer in which cancer cells do not express either estrogen or/progesterone receptors, and also do not synthesize small or higher amounts of human epidermal growth factor receptor 2 (HER2) [91,92]. The primary mechanism of action of Dox in chemotherapy results in the molecular interactions of the drug with cellular topoisomerase II, and deoxyribonucleic acid (DNA), thus leading to DNA damage, and inhibition of ribonucleic acid (RNA) synthesis [93,94]. Despite the fact that Dox possesses effective therapeutic efficacy in many medical conditions and alignments, Dox still poses long term side effects which includes, dose-related progressive cardiomyopathy, cough or hoarseness accompanied with fever, and joint pains etc. [95]. In order to reduce these side effects, Dox may be encapsulated with liposomes that are loaded with nonsteroidal anti-inflammatory drugs such as celecoxib [90,96]. The above-mentioned drug encapsulation framework could also be employed in the synthesis of non-toxic and eco-friendly liposomal NPs loaded with therapeutic agents (e.g., photosensitizing, antibacterial, or anti-inflammatory drugs). For example, co-encapsulated sodium diethyldithiocarbamate and zinc phthalocyanine loaded in liposomes displayed a higher phototoxicity against human breast cancer (MDA-MB 231) cells [97]. Furthermore, biodegradable plasmon resonant liposome Au NPs resulted in complete ablation of tumor mass post-irradiation of mouse tumor xenograft model using a 750 nm laser [98]. A schematic illustration of the green synthesised AgNPs encapsulated with liposomes that are loaded with a photosensitizing agent, zinc phthalocyanine tetrasulfonic acid (ZnPcS_4_), and the characterization techniques are shown in Figure 4.

### 4.2. Functionalization of Encapsulated NPs

**Figure 5:**
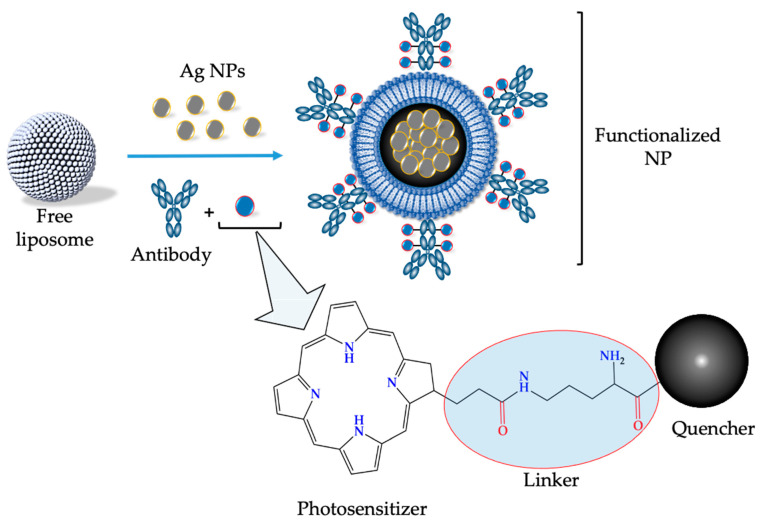
Schematic representation of hybridized liposome encapsulated silver nanoparticles (Ag NPs) functionalized with activatable PS.

Complementary investigations have established that surface functionalization of NPs for therapeutic use has greater advantages when compared to non-surface functionalized NPs. This is because functionalized NPs can be characterized by higher stability, selectivity, and drug delivery efficiency [64]. In order to overcome certain limitations associated with unfunctionalized NPs such as high cost, lack of specificity, and increased toxicity, there are different functionalization approaches employed in nanotechnology. Antibody mediated targeting is alternatively one of the most common and efficient approach for active targeting [98]. When functionalized with targeting ligands/ antibodies, NPs can target specific cancer cell receptors thus, making it easier for therapeutic components to accumulate within targeted tumor cells. Conceptually, the conjugation process of ligands/ antibodies onto the surface encapsulated liposomal NPs involves the fabrication of active targeting moieties with highly concentrated linking molecules (e.g., peptide linker) [3,56]. In most cases, these targeting moieties (e.g., monoclonal antibodies, folic acid, transferrin, and carbohydrates) interact with extracellular receptor present on the surface of many cancer cells [99]. Alternatively, it is obvious that improving the physical and chemical properties of encapsulated NPs could enhance the desired therapeutic effects of PDT [56,100]. For example, the encapsulation of green synthesized metallic NPs and activatable inorganic nano-formulations as PSs could reduce some of the therapeutic limitations associated with conventional PSs (Figure 5).

## 5. Green Hybridized Activatable NPs in PDT

Despite the fact that PDT offers several advantages over conventional therapies such as radiotherapy and chemotherapy, PDT has its own drawbacks e.g., induction of acute inflammatory reactions [101]. Due to other restrictions such as light penetration depth and the difficulty of propagating light into tissue, PDT is currently an underutilized clinical treatment therapeutic option that is mostly employed in the treatment and management of superficial tumors [101,102]. In addition to the above-mentioned limitations, conventional PSs used for therapeutic purposes are always in an “on” state thus, limiting their application in clinical studies [102]. Interestingly, the development of hybridized activatable NPs that can only be turned “on” when hybridized activatable NPs are exposed to different cellular conditions e.g., pH, or their interactions with enzymes, amino acids, and visible-to-NIR-light plays a vital role in active targeting of cancerous cells [103,104]. 

**Figure 6:**
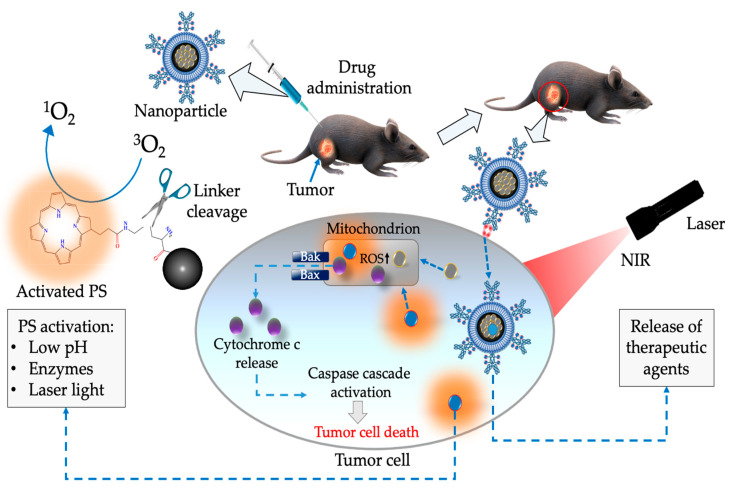
A schematic diagram illustrating the administration and therapeutic effects of functionalized nanoparticles in cancer PDT.

As represented in Figure 6, multi functionalized NPs with targeting moieties and therapeutic agents can selectively target tumor cells and induce cell death. The therapeutic effects of this multi functionalized complex can inhibit tumor cell proliferation via the upregulation of BH3-only pro-apoptotic proteins (e.g., BAK/BAX) or/down regulation of anti-apoptotic proteins such as A1/BFL-1,BCL-XL, BCL-W, BCL-2, and MCL-1 [105,106]. Once BH3-only pro-apoptotic proteins (i.e., BAK/BAX) oligomerize onto the mitochondrial outer membrane which eventually leads to mitochondrial outer membrane permeabilization [105,107]. After permeabilization of the mitochondrial outer membrane, haemoprotein cytochrome c gets released into the cytoplasmic matrix of the cell. Within the cytoplasmic matrix, cytochrome c may interact with cytoplasmic proteins e.g., apoptotic protease activating factor 1 (APAF 1) that plays a significant role in caspase cascade activation [108,109]. Furthermore, the generation of ROS within intracellular organelles such as the mitochondria, lysosomes, and the nucleus is reported of causing oxidative damage on cellular macromolecules e.g., proteins, enzymes, ribonucleic acids (RNA), deoxyribonucleic acid (DNA), and lipids such as plasma membrane phospholipids [110].

### Limitations of Green Synthesized Metallic NPs

The synthesis of green metallic nanomaterials has received tremendous attention over the past decade [111]. Like any other therapy, the therapeutic applications of green NPs are still questionable. This is because there is still little or/inadequate information on how these nanostructures interact with various intracellular organelles and proteins [112,113]. Other challenges of the use of green NPs for therapeutic purposes includes the availability of raw materials, different reaction conditions, and control of particle size, shape, and optical properties [114]. These factors have continued to hinder the development of green nanomaterials at a large scale. Green NP synthesis may also require the use of optimized temperatures, as a result, this may lead to high energy consumption [115]. Another barrier that could be associated with the synthesis of green nanomaterials is the lack of standardized protocols that can be used to control the diameter, shape, stability, optical properties, bioaccumulation, and toxicity [116,117]. In addition, recent studies have reported variations in particle size and shape of green NPs [115,118]. These variations have also been reported to confirm with ideal optical properties of NPs [117]. In order to avoid some of these shortcomings, current studies are now focused on trying to reduce dose-dependence in conventional therapies (e.g., chemotherapeutic drugs such as Doxorubicin) with eco-friendly green NPs, or/other nanomaterials [119].

## 6. Conclusions, Outlook, and Future Perspectives

Although conventional therapies are being used in the treatment or/management of various medical complications, their clinical application in certain medical conditions is still restricted. One of the major setbacks that limits the therapeutic applications of conventional therapies may include poor aqueous stability, lack of specificity, higher dosage and toxicity, multidrug resistance, and tumor recurrence. Out of these therapeutic limitations of conventional therapies, multidrug resistance in cancer therapy is one of the most common limitations associated with chemotherapy. This has led to a continual search for relatively safe alternative and complementary therapeutic approaches where green nanotechnology is a promising candidate. Over the past decade, green synthesized NPs have been widely used in in vitro studies. Additionally, there is insufficient or little information about the therapeutic potential of green synthesized metallic NPs in in vivo and clinical studies. Therefore, this review presents therapeutic information and potential of eco-friendly biocompatible green synthesized NPs for PDT of cancer. The current review also gives insights on the synthesis, functionalization of green NPs, and their probable underlying mechanisms post treatment. Considering the evaluations and therapeutic potential of green synthesized NPs in cancer treatment, it is necessary to conduct more research to understand the long-term chemical and physical characteristics of green NPs and their molecular interactions post-treatment. 

## Figures and Tables

**Table 1:** Summarizes the advantages and disadvantages of photodynamic therapy and other conventional therapies for cancer treatment [6,7,8,9,10,11].

Therapeutic Options	Classification	Advantages	Disadvantages
Photodynamic therapy	Localized	Non-invasive, short treatment time, can be targeted, can be repeated, immunogenic, with fewer adverse effects post-treatment, and cost-effective	Photosensitivity and sun-shade post-treatment, limited light penetration, photosensitizer, and molecular oxygen dependent
Surgery	Localized	Quick and effective, light independent, improves the quality of life, and cost-effective when compared to other systemic therapies	Invasive, painful, wound bleeding, numbness, susceptibility to infections, swelling, tissue injury, loss of organ function/or body organ, and induction of secondary metastatic tumors
Radiotherapy	Localized	Non-invasive when compared to surgery, low toxicity when compared to systemic therapies e.g., chemotherapy and hormone therapy, cost-effective, with greater accessibility, and convivence	Unsuitable for systemic cancers, increased need for imaging techniques such as magnetic resonance imaging (MRI), limited information on adverse effects, greatest chances of inducing the development of secondary cancers
Chemotherapy	Systemic	Can reach malignant cells in all body sites, light independent, suitable for systemic cancers such as leukemias, testicular cancers, and lymphomas	Increased systemic toxicity, induction of multidrug resistance (MDR), hair loss, weight loss, induction of fertility complications, and can lead to peripheral neuropathy /or other nervous system complications e.g., numbness

**Table 2:** Summary and classification of photosensitizers used in clinical studies [12,13,14,15,16,17,18].

Trade Name/ Photosensitizer	Generation	λ_max_ (nm)	*ε*_max_ (M^−1^ cm^−1^)	^1^O_2_ Quantum Yield (Φ_Δ_)	Current Indications and Clinical Applications
Photofrin^®^ (HpD, Porfimer sodium)	First	630	3.0 × 10^3^	~0.01	Bowen’s disease, bladder, brain, breast, cutaneous Kaposi’s sarcoma, cervical, and lung cancers
Foscan^®^ (m-THPC, Temoporfin)	Second	652	3.0 × 10^4^	0.43	Advanced head and neck cancers
Visudyne^®^ (Verteporfin)	Second	686	3.4 × 10^4^	0.7	Subfoveal choroidal neovascularization
Photochlor^®^ (HPPH, 2-(1-hexyloxyethyl)-2-devinyl pyropheophorbide-alpha)	Second	665	4.75 × 10^4^	0.48	Basal cell carcinoma, Barrett’s esophagus, non-small lung, and esophageal cancers
Levulan^®^ (5-Aminolevulinic acid)	Second	635	5 × 10^3^	0.56	Basal cell carcinoma, brain, skin, bladder, and head and neck cancers
Lutrin^®^ (Lutetium texaphyrin)	Second	732	4.2 × 10^4^	0.11	Kaposi’s sarcoma, melanoma, cervical, prostate, and breast cancers
Tookad^®^ (Palladium-bacteriopheophorbide)	Second	762	8.85 × 10^4^	0.50	Prostate cancer
Photosens^®^ (Sulfonated aluminum phthalocyanines)	Second	675	20 × 10^4^	0.38	Breast, cervical, skin, lung, and head and neck cancers
Purlytin^®^ (Tin ethyl etiopurpurin)	Second	664	3 × 10^4^	0.7	Basal cell carcinoma, breast, and Kaposi’s sarcoma
Laserphyrin^®^ (Mono-L-aspartylchlorin-*e*_6_)	Second	654	4.0 × 10^4^	0.77	Liver, lung, and head and neck cancers

**Table 3:** Classification and characteristics of nanomaterials used in cancer therapy.

Nanomaterial Classification	Nanomaterial	Size (nm)	Physicochemical Properties	Medical Applications	Ref.
Inorganic nanoparticles (INPs)	Quantum dots	2–10	Optoelectronic, higher surface-to-volume ratio, narrow emission spectra, higher quantum yield, and good biocompatibility	Drug delivery systems, bioimaging, biosensing, and PDT	[31,32,33]
	Carbon-based NPs	<10	Optoelectronic, water soluble, higher light absorption coefficient, biocompatibility, and stable chemical inertness, with excellent photon induced electron transfer	Drug delivery systems, bioimaging, biosensing, and PDT	[34,35,36,37,38]
	Ceramic NPs	<50	Optoelectronic, corrosion-resistant, higher biocompatibility, and heat resistance	Drug delivery systems, bioimaging, and PDT	[39,40,41]
	Gold NPs	88–252	Optoelectronic, higher atomic number, localized surface plasmon resonance with a higher X-ray absorption coefficient, and can easily be functionalized with other targeting are moieties	Drug delivery systems, bioimaging, biosensing, radiotherapy and PDT	[42,43,44,45,46]
	Silica NPs	50–100	Optoelectronic, higher stability and biocompatibility, with a large surface area that can easily be functionalized with other targeting are moieties	Catalysts, drug delivery systems, bioimaging, biosensing, and PDT	[47,48,49,50]
Organic nanoparticles (ONPs)	Dendrimers	2–15	Multivalent surface, low polydispersity, chemically stable, self-assembling, good biocompatibility, and easily functionalized with other targeting are moieties	Drug delivery systems, bioimaging, biosensing, neutron capture therapy, and PDT	[39,45,51]
	Liposomes	~50	Consist of one or more phospholipid bilayers, highly biocompatible, with minimized cellular toxicity, and can easily be functionalized with other targeting are moieties	Drug delivery systems, bioimaging, biosensing, diagnostics, theranostic, and PDT	[52,53]
	Micelles	~20	Polar heads and non-polar tails, with high loading capacity, good biocompatibility with minimized cellular toxicity, and can easily be functionalized with other targeting are moieties	Drug delivery systems, bioimaging, biosensing, theranostic, and PDT	[54,55,56]
	Ferritin	9.5–32.3	Composed of 24 protein subunits with mass ranging 450–500 kDa, optoelectronic, chemically stable, highly biocompatible, and can easily be functionalized with other targeting are moieties	Drug delivery systems, bioimaging, biosensing, theranostic, and PDT	[57,58]

**Table 4:** Common medicinal plants used in the synthesis of metallic NPs for therapeutic use.

Plant Name	Plant Part	Metal	NP Size (nm)	λ (nm)	Activity	Ref.
*Rubus fairholmianus*	Roots	Ag	~30–150	455	Anticancer activity against human breast cancer (MCF-7) cells	[72]
*Acalypha indica*	Leaves	Ag	20–30	420	Antibacterial activities against *Escherichia coli* (*E. coli*), and *Vibrio cholerae*	[73]
*Artemisia vulgaris*	Leaves	Au	50–100	544	Larvicidal activity against dengue fever vector *Aedes aegypti* L.	[74]
*Iresine herbstii*	Leaves	Ag	44–64	438	Anticancer activity against human cervical cancer (HeLa) cells	[75]
*Allium sativum*	Bulbs	Ag	20–40	452	Anticancer activity against human lung epithelial (A549) cells	[76]
*Abutilon indicum*	Leaves	Ag	1–300	455	Anticancer activity against colon carcinoma (COLO 205) cells, and antibacterial effects against *Bacillus cereus*, *E. coli*, *Salmonella typhi*, *Staphylococcus aureus*, *Shigella flexneri*, and *Pseudomonas fluorescence*	[77]
*Annona squamosa*	Leaves	Ag	20–100	444	Anticancer activity against human breast cancer (MCF-7) cells	[78]
*Artocarpus hirsutus*	Leaves	Au	5–40	540	Anticancer activities against colon carcinoma (RKO), Hela, and A549 cells	[79]
*Curcuma wenyujin*	-	Au	530	200	Anticancer activity against human renal cancer (A498) cells	[80]
*Nerium oleander*	Stem bark	Au	10–100	534–553	Anticancer activity against human breast cancer (MCF-7) cells	[81]
*Sargassum swartzii*	Whole plant	Au	154	525	Anticancer activity against pancreatic cancer (PANC-1) cells	[82]
*Lonicera japonica*	Leaves	Ag	-	456	Anticancer activity against human lung epithelial (A549) cells	[83]
*Spinacia oleracea L*	Leaves	Au	16.7	549	Anticancer activities against endometrial cancer (HEC-1-A, HEC-1-B, Ishikawa, and KLE) cell lines	[84]
*Taxus baccata*	Needles	Au	20	300–400 and 500–600	Anticancer activities against MCF-7, Hela, and ovarian (Caov-4) cell lines	[85]

## Data Availability

Not applicable.
